# Left Ventricular-Arterial Coupling and Vascular Function in Childhood Cancer Survivors Exposed to Anthracycline Chemotherapy

**DOI:** 10.31083/j.rcm2404124

**Published:** 2023-04-23

**Authors:** Massimiliano Camilli, Lisa Birritella, Angelica Bibiana Delogu, Priscilla Lamendola, Antonio De Vita, Veronica Melita, Alberto Romano, Antonio Ruggiero, Giorgio Attinà, Gaetano Antonio Lanza, Massimo Massetti, Filippo Crea, Antonella Lombardo

**Affiliations:** ^1^Department of Cardiovascular and Thoracic Sciences, Fondazione Policlinico Universitario A. Gemelli IRCCS, 00168 Rome, Italy; ^2^Department of Cardiovascular and Pulmonary Sciences, Catholic University of the Sacred Heart, 00168 Rome, Italy; ^3^Catholic University of The Sacred Heart, 00168 Rome, Italy; ^4^Unit of Pediatrics, Pediatric Cardiology, Department of Woman and Child Health and Public Health, Fondazione Policlinico Universitario A. Gemelli IRCCS, 00168 Rome, Italy; ^5^Pediatric Oncology Unit, Department of Woman and Child Health and Public Health, Fondazione Policlinico Universitario A. Gemelli IRCCS, 00168 Rome, Italy

**Keywords:** childhood cancer survivors, cardio-oncology, echocardiography, left ventricular-arterial coupling, endothelial function

## Abstract

**Background:**

Cardiovascular (CV) diseases are a cause of increased long-term morbidity and mortality in childhood cancer survivors (CCSs) treated with anthracyclines. These drugs may affect not only the heart, but also the vascular system. Left ventricular-arterial coupling (LVAC) represents a reliable parameter of altered ventricular and vascular performance, with validated prognostic value and never investigated in this setting. Aim of this study was to assess, in CCSs and matched controls, LVAC changes, performed with different echocardiographic modalities, and their relationship with endothelial function.

**Methods:**

Twenty survivors treated with anthracyclines for childhood malignancies and a matched control group of 20 healthy subjects were enrolled. Arterial elastance (Ea), end-systolic elastance (Ees), Ea/Ees ratio, as well as three-dimensional (3D) LVAC (assessed by measurement of End Systolic Volume [ESV]/Stroke Volume [SV] ratio) were performed at rest. Endothelial function was evaluated by measurement of flow-mediated dilatation (FMD) of the brachial artery.

**Results:**

3D SV and 3D ESV/SV ratio resulted respectively significantly lower and higher in CCSs than in controls, while Ea, Ees and Ea/Ees ratio were not different among groups. A positive correlation between 3D ESV/SV ratio and cumulative anthracycline doses, as well as with time after drug exposure were also found. Mean FMD was similar in CCSs and controls (8.45 ± 1.79 versus 9.41 ± 3.41, *p* = 0.34).

**Conclusions:**

In conclusion, conventional LVAC parameters were not shown to be significantly different between CCSs and controls; however, 3D SV and LVAC were significantly impaired in our population. In these patients, endothelial function was comparable to controls. Larger validation studies are therefore needed.

## 1. Introduction 

Childhood cancer prognosis has significantly improved in the last decades, thanks to earlier diagnosis and more effective treatments [1, 2]. The increase in survival rates, however, has also resulted in long-term sequelae due to anti-neoplastic drugs [1]. Cardiovascular (CV) diseases, in particular heart failure (HF), represent a leading cause of non-oncologic morbidity and mortality in this highly-vulnerable population [3, 4]. Anthracyclines, used in a great variety of hematological and solid pediatric malignancies, are associated with cardiomyocyte damage, that can lead to impaired cardiac function, even decades after chemotherapy [4, 5]. Moreover, it is well known that childhood cancer survivors (CCSs) are at increased risk of hypertension, diabetes and obesity [6, 7], which may negatively impact on an already fragile system and predispose to CV diseases.

The early detection of subclinical left ventricle (LV) dysfunction can be crucial in these patients, as it might promote a closer clinical follow-up and treatment aimed to prevent or reduce the evolution toward overt LV dysfunction and HF. At present, however, no reliable marker of risk of LV dysfunction has been identified [4, 5, 8]. In a previous paper on the same population, our group demonstrated that three-dimensional (3D) LV ejection fraction (LVEF) was able to detect subclinical changes of ventricular function in CCSs [9].

Although CV risk assessment has traditionally focused on the heart, vascular dysfunction may also occur and persist for years after chemotherapy. However, whether vascular alterations happen and influence cardiac function in CCSs remains poorly investigated. Left Ventricular-Arterial Coupling (LVAC) is becoming a valid surrogate of myocardial mechanics, integrating arterial load and LV end-systolic elastance, hence quantifying chamber stiffness and contractility [8, 9, 10]. It provides an assessment of CV performance and efficiency, with higher values of LVAC ratio reflecting a compromised ventricular-vascular matching [10, 11, 12]. Importantly, LVAC has been found to have prognostic value in HF adult patients [10, 11, 12] but, despite its importance, it has never been investigated in CCSs previously treated with anthracyclines.

Aim of this study was to assess LVAC, performed with different echocardiographic modalities, as well as vascular function, reported as flow mediated dilatation (FMD), in CCSs and matched controls.

## 2. Materials and Methods

### 2.1 Study Population

Twenty survivors of childhood cancer treated with anthracyclines were enrolled to receive a cardiac long-term follow-up [7]. Inclusion criteria were: (1) previous doxorubicin/epirubicin exposure; (2) a minimum follow-up of at least 3 years; (3) cumulative anthracycline dose <360 mg/$m^{2}$; (4) no symptoms of HF and normal global LV systolic function [defined as a 3D LV ejection fraction (LVEF) ≥55%, and two-dimensional LV shortening fraction (LVSF) ≥28%] at standard echocardiogram before chemotherapy initiation; (5) no CV risk factor at baseline. Exclusion criteria included a history of congenital cardiac defects; more than mild valvular heart defects or other cardiac diseases; mediastinal/chest radiotherapy; bone marrow or stem cells transplantation; development of second neoplasm necessitating chemotherapy at follow-up.

From October 2019 to February 2020, 48 patients affected by pediatric malignancies and exposed to anthracyclines were scheduled to have a visit at the Children Cancer Survivor Service of the Pediatric Oncology Unit at “Policlinico Universitario Agostino Gemelli” in Rome. Of them, 15 were excluded because underwent chest radiation. 4 cases had developed secondary malignancies requiring other chemotherapy regimens. 29 survivors were eligible for the study: however, 4 of them did not accept to participate and 5 had inadequate echocardiographic window for full echocardiographic examination. Thus, 20 subjects treated with anthracyclines were finally enrolled in the study.

All patients received anthracycline therapy by a central line, with infusion lasting 4 h. The doxorubicin-equivalent doses were calculated according to the previously published equivalence ratio: epirubicin (× 0.67) [13].

Twenty healthy non-athletic subjects comparable for age, sex and body surface area were also enrolled as a control group. Demographic and clinical characteristics were collected from all subjects; malignancy and chemotherapy related information were collected for CCSs patients.

The study was performed in accordance with the Helsinki declaration; all subjects provided written informed consent. The study was approved by the local Ethics Committee; the approval number is DIPUSVSP-15-11-2239.

### 2.2 Echocardiography

All subjects underwent transthoracic two and three-dimensional (2D/3D) echocardiography, together with Tissue Doppler (TDI) and speckle tracking (ST) imaging, using a standard commercial ultrasound machine (Philips EPIQ7C machine, X5-1 Transducer, Philips Medical Systems, Andover, Massachusetts, USA). Routine 2D images were acquired as recommended [14]. LV function was evaluated through LVEF, obtained using Simpson biplane formula and 3D echo dataset. 2D-ST analysis with global longitudinal strain (GLS) calculation was obtained in the entire population.

Arterial elastance (Ea) was calculated as the ratio of end-systolic pressure (ESP) to 2D stroke volume (SV) [8]. ESP was determined by the validated equation 0.9 × systolic blood pressure (SBP) measured by manual sphygmomanometry [10]. End-systolic elastance (Ees) was calculated using the validated single beat technique, through the measurements of blood pressure, SV, LVEF, and pre-ejection and systolic ejection time intervals from LV outflow Doppler. 3D LVAC was instead estimated as the 3D end-systolic volume (ESV) to 3D LV stroke volume (SV) ratio [10]. 3D measurements of LV volumes, LVEF and SV have been proved to be more accurate/reproducible compared to standard 2D echocardiography and similar to those obtained with cardiac magnetic resonance (CMR) [15, 16].

All digital loops were acquired by a cardiologist, stored in a workstation and interpreted by a cardiologist, expert in non-invasive imaging, together with a pediatric cardiologist, who were blind to the subject’s group.

Intra-observer variability of 2D/3D LVEF was assessed by one reader (M.C.) analyzing 2D/3D LVEF in 10 echocardiograms twice. Inter-observer variability was assessed by two readers (M.C. and P.L.) analyzing the same 10 echocardiograms and the same parameters.

Cardiotoxicity was defined as LVEF decline below the lower limit of normal, which was considered as 50%, in line with the definition reported in current guidelines [17, 18].

### 2.3 Assessment of Endothelial Function

As extensively described in previous reports, endothelium-dependent arterial dilator function was studied by measuring flow-mediated dilatation (FMD) at the brachial artery level [19, 20, 21]. Briefly, the patient remained supine for at least 10 minutes with the left brachial artery displayed on a high-resolution ultrasound system (Artida; Toshiba, Milan, Italy) by a high frequency vascular probe (10 MHz). A dedicated software should be connected to the echo machine. The arterial segment to be analyzed (region of interest) should be selected by the operator and the system software automatically identifies the vessel’s inner edges and traces the artery walls using the different echogenicity with the lumen. Afterwards, measures the brachial artery diameter and Doppler blood flow velocity are automatically elaborated during the whole duration of the test without any operator’s intervention and with the probe maintained in a fixed position by a mechanical support. After acquisition of baseline images of the brachial artery for one minute, a sphygmomanometer cuff is inflated to 200 mmHg to induce forearm ischemia; the cuff is released after 5 minutes to elicit forearm reactive hyperemia and the brachial artery diameter is monitored for 5 minutes. At the end, FMD is calculated as the maximum percentage change of the brachial artery diameter recorded during hyperemia compared to basal conditions.

### 2.4 End-Points

The primary endpoint of the study was the detection of differences in terms of LVAC and FMD between CCSs and controls. Secondary endpoints were: (1) evaluation of the correlation between LVAC values and anthracycline doses, as well as years after drug exposure. Moreover, (2) whether FMD values were related to LVAC changes and anthracycline doses.

### 2.5 Statistical Analysis

Continuous variables are reported as means and standard deviations, while categorical variables as numbers and percentages. The analysis of variance was used to compare continuous variables, whereas nominal variables were compared by Fisher exact test. Correlation analysis was done by Pearson’s test. Statistical significance was set at a *p* value < 0.05. Statistical analyses were performed using the Statistical Package for Social Sciences, version 23.0 (SPSS, Chicago, IL, USA).

## 3. Results

### 3.1 Study Population

Demographic and clinical characteristics of patients and controls were previously reported in another paper [9] and synthetized in Table 1.

**Table 1.** **Clinical Features and Echocardiographic characteristics of Children Cancer Survivors and healthy subjects**.

	CCSs	Healthy Controls	*p*-value
Age, years ± SD	13.2 ± 2.8	12.4 ± 2.9	0.410
Male sex, n (%)	11 (55%)	13 (65%)	0.531
Weight, kg ± SD	50.9 ± 15.2	56.1 ± 18.7	0.419
Height, cm ± SD	155.0 ± 15.6	156.7 ± 18.8	0.800
BSA, m² ± SD	1.4 ± 0.3	1.5 ± 0.3	0.610
Heart rate, bpm ± SD	82.9 ± 9.9	79.9 ± 11.3	0.427
SBP, mmHg ± SD	108.0 ± 5.3	109.1± 6.6	0.636
DBP, mmHg ± SD	63.1± 2.6	63.4 ± 3.4	0.811
2D EDV, mL ± SD	70.3 ± 19.9	78.0 ± 26.9	0.383
2D ESV, mL ± SD	25.1 ± 8.4	27.9 ± 12.2	0.474
2D LVEF, % ± SD	64.3 ± 5.0	64.3 ± 4.5	1
3D EDV, mL ± SD	73.5 ± 26.0	84.1 ± 30.1	0.328
3D ESV, mL ± SD	26.6 ± 9.9	25.8 ± 11.7	0.832
3D LVEF, % ± SD	63.9 ± 3.9	69.8 ± 5.7	**0.002**
3D SV, mL ± SD	45.6 ± 5.8	56.3 ± 18.4	**0.015**
IVCT (ms)	79.8 ± 16.5	67.9 ± 9.5	**0.046**
ET (ms)	277.4 ± 28.7	290.4 ± 19.5	0.214
Ea, mmHg/mL ± SD	2.3 ± 0.7	2.3 ± 1.5	1
Ees, mmHg/mL ± SD	3.1 ± 1.0	3.2 ± 1.8	0.822
LVAC 2D Ea/Ees	0.8 ± 0.1	0.7 ± 0.1	0.227
LVAC 3D ESV/SV	0.7 ± 0.2	0.4 ± 0.1	< **0.001**

**Abbreviations**. 2D, Two dimensional; 3D, Three dimensional; BSA, Body Surface Area; CCSs, Children Cancer Survivors; DBP, Diastolic Blood Pressure; Ea, arterial elastance; Ees, end-systolic elastance; EDV, end diastolic volume; ESV, end systolic volume; ET, ejection time; IVCT, isovolumic contraction time; LVAC, left ventricular-arterial coupling; LVEF, left ventricular ejection fraction; SBP, Systolic Blood Pressure; SD, standard deviation; SV, stroke volume. In bold, significant results.

Briefly, age, sex and other main clinical characteristics were similar in the two groups. Mean age of CCS patients was 13.2 ± 2.8 years and 11 (55%) were male; the median age at the time of diagnosis and at the beginning of cancer treatment was 9.0 years (±11.9 years). The mean cumulative doxorubicin isotoxic equivalent dose was 234.5 ± 87.4 mg/$m^{2}$ and the mean follow-up time was 6.5 ± 2.7 years. Echocardiographic parameters are also reported in Table 1. Table 2 shows oncologic features of CCS. During follow-up, no patient reported significant symptoms or showed signs of cardiac disease at physical examinations. Finally, no evidence of cardiac toxicity was reported in survivors [17, 18].

**Table 2.** **Main Cancer Survivors’ Characteristics relative to neoplastic history**.

Specific Charateristics of CCS goup.	
Age at diagnosis, years ± SD	9.0 ± 11.9
Years since last anthracycline dose, months ± SD	6.5 ± 2.7
Cumulative anthracycline dose, mg/$m^{2}$ ± SD	234.5 ± 87.4
Acute lymphoblastic leukemia, n (%)	8 (40)
Hodgkin lymphoma, n (%)	3 (15)
Non-Hodgkin lymphoma, n (%)	3 (15)
Ewing sarcoma, n (%)	5 (25)
Neuroblastoma, n (%)	1 (5)

**Abbreviations**. CCS, childhood cancer survivors; n, number; SD, standard deviation.

### 3.2 Ventricular-Arterial Coupling

**Fig. 1.**
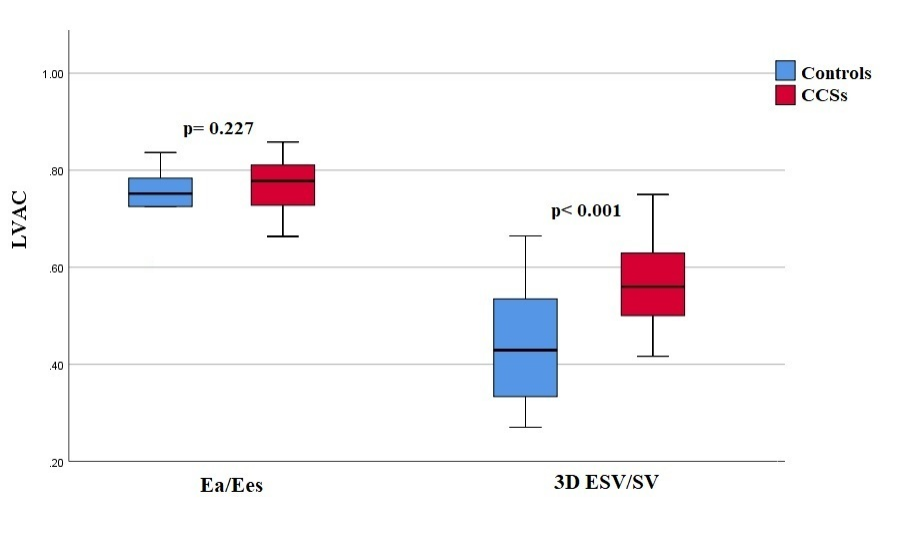
**Box Plots representing Left Ventricular-Arterial Coupling, expressed as Ea/Ees Ratio and as 3D ESV/SV Ratio, between Childhood Cancer Survivors and Controls**. Abbreviations: CCS, Childhood Cancer Survivors; Ea, Arterial Elastance; Ees, End-Systolic Elastance; ESV, End Systolic Volume; LVAC, Left Ventricular-Arterial Coupling; SV, Stroke Volume.

Ea and Ees values were not significantly different among studied groups (respectively 2.3 ± 0.7 mmHg/mL in CCSs versus 2.3 ± 1.5 mmHg/mL in controls, *p* = 1.0 and 3.1 ± 1.0 mmHg/mL versus 3.2 ± 1.8 mmHg/mL, *p* = 0.822). At the same time, Ea/Ees ratio was similar in CCSs and patients not previously exposed to anthracyclines (0.8 ± 0.1 versus 0.7 ± 0.1, *p* = 0.227) (Fig. 1). In the CCSs population, Ea/Ees did not show any significant correlation with cumulative anthracycline dose (*p* = 0.130) and/or years after last drug administration (*p* = 0.530). 3D SV was significantly lower in CCSs than controls (45.6 ± 5.8 mL versus 56.3 ± 18.4 mL, *p* = 0.015), while mean 3D ESV/SV ratio values were significantly higher in the cancer population (0.67 ± 0.18 versus 0.44 ± 0.11, *p *< 0.001) (Fig. 1).

**Fig. 2.**
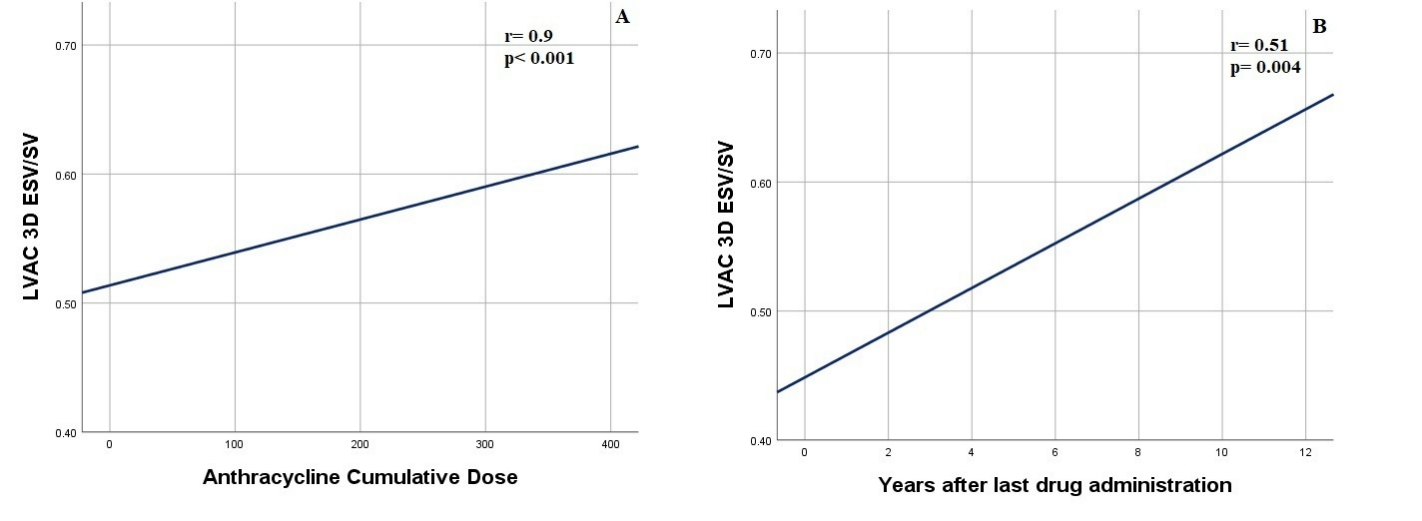
**Correlation between Three-Dimensional Left Ventricular-Arterial Coupling and Cumulative Anthracyline Dose (A) and Years after last drug administration (B)**. Abbreviations: ESV, End Systolic Volume; LVAC, Left Ventricular-Arterial Coupling; SV, Stroke Volume.

Moreover, a statistically significant positive correlation between the 3D ESV/SV ratio and the cumulative anthracycline dose (Fig. 2A), as well as with time after drug exposure (expressed in years) (Fig. 2B) were found (r = 0.9, *p *< 0.001 and r = 0.51, *p* = 0.004).

### 3.3 Endothelial Function, LV-Arterial Coupling and Anthracycline Cumulative Dose

**Fig. 3.**
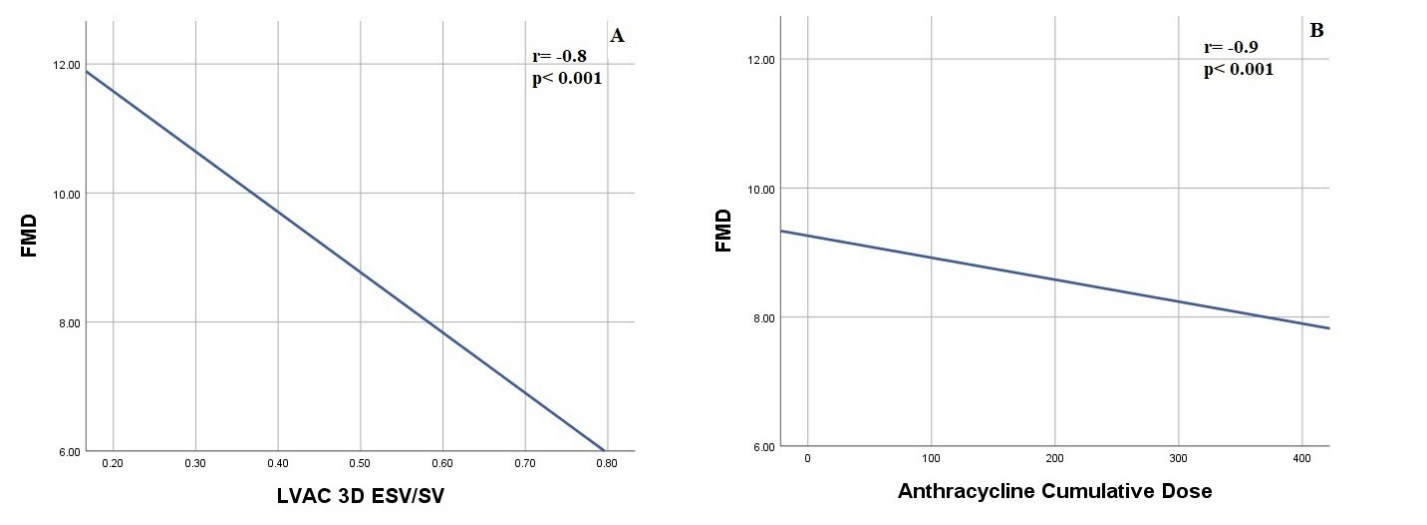
**Correlation between Endothelial Function, expressed as Flow Mediated Dilatation, and Three-Dimensional Left Ventricular-Arterial Coupling (A) and Cumulative Anthracyline Dose (B)**. Abbreviations: ESV, End Systolic Volume; FMD, Flow Mediated Dilatation; LVAC, Left Ventricular-Arterial Coupling; SV, Stroke Volume.

No difference in FMD values was found between CCS patients and controls (8.4 ± 1.8 versus 9.4 ± 3.4%; *p* = 0.340). When evaluating the CCSs group only, we could neither identify a significant correlation between FMD values and Ea, Ees and Ea/Ees. However, an inverse correlation between FMD values and 3D ESV/SV (r = –0.8, *p *< 0.001) was observed (Fig. 3A), as well as between FMD and anthracycline cumulative dose (r = –0.9; *p *< 0.001) (Fig. 3B).

## 4. Discussion

To our knowledge, this is the first study that extensively reports, through multiple echocardiographic modalities, LVAC in CCSs previously treated with anthracyclines, relating these parameters with endothelial function assessed by FMD. Several findings hence emerge from our investigation: (1) Ea, Ees and Ea/Ees did not differ between CCSs and controls; (2) 3D SV was significantly lower in CCSs; (3) 3D ESV/SV ratio was significantly higher in CCSs; (4) lack of remarkable difference between survivors and controls was detected in terms of endothelial function; (5) among CCSs, 3D LVAC parameters showed a significant correlation with FMD values, anthracycline cumulative doses, as well as with years after drug exposure.

The population of cancer survivors is increasing over time. This has been paralleled by the growing evidence of long-term adverse effects of cancer therapies, the need for a better understanding of the mechanisms underlying CV toxicities and early identification of these negative effects [4, 22, 23]. Anthracycline cardiotoxicity may occur early after the first cycles or become manifest even several years later, highlighting the necessity of detecting subgroups of patients at increased risk of developing HF [4, 22, 23]. However, in cancer survivors’ programs, evaluation of cardiac function is almost exclusively assessed through LVEF [1].

Non-invasive measurement of LVAC is feasible in children [24], may provide comprehensive assessment of LV performance and has been demonstrated to give incremental information to LVEF in the characterization and management of patients with HF, pulmonary hypertension and coronary artery disease (CAD) [10]. In the setting of cardio-oncology, however, evidence on LVAC remains sparce. Narayan *et al*. [25] showed, in 135 adult patients affected by breast cancer treated with anthracyclines, that LVAC alteration may precede LVEF deterioration [26]. Moreover, a recent study demonstrated that LVAC decreases early after anthracycline exposure, primarily because of a change in LV elastance relative to arterial elastance [27]. In a further report on breast cancer patients previously exposed to chemotherapy, LVAC was not altered at rest, but it was impaired during exercise echocardiography at 7 years of follow-up [28].

Long-term effects of anthracyclines on LVAC have never been investigated in a highly vulnerable population as survivors of pediatric malignancies. In these subjects, we found no significant differences neither in terms of arterial and ventricular elastance assessed with 2D echocardiography, nor relating to their ratio. On the other hand, lower 3D stroke volume was detected in CCSs. As a consequence, significantly higher 3D ESV/SV values were observed in our population than in controls, with a positive significant correlation between 3D LVAC and both anthracycline cumulative doses administered and time after exposure.

These findings merit further discussion: SV at rest was reduced in a limited group of breast cancer survivors almost 10 years post-anthracycline chemotherapy completion and this alteration was conceivably responsible for impaired exercise capacity in these patients [29]. In our report, the reduction in 3D SV, which assessment is feasible and accurate both in aldults and in the pediatric population [30], may corroborate the alteration of 3D LVAC; moreover, this last metric is also a surrogate of 3D LVEF, which in a previous paper on the same population [9], resulted to be the only parameter significantly altered between groups.

Yet, in this study we failed to detect marked differences in endothelial function, as assessed by FMD. Endothelial dysfunction is an early step in the pathogenesis of many CV diseases, in particular atherosclerosis, and plays an important role in their development and progression [21]. Of importance, endothelial dysfunction has been suggested to participate in the pathogenesis of myocardial damage related to anthracycline therapy, possibly mediated by increased oxidative stress, decreased NO production and increase in NO inactivation [28]. Besides these solid basis, previous studies showed contrasting data on the effects of anthracycline therapy on vascular endothelial function, as assessed by FMD, probably because of differences in anthracycline doses administered, follow-up times and coexistence of traditional CV risk factors [31, 32, 33, 34, 35]. Our data demonstrated that, in this relatively small cohort, endothelial-dependent function appears normal in the years after treatment, suggesting an inherent endothelial plasticity after removal of the toxic perturbation.

Significant negative correlations between FMD, 3D ESV/ESV and anthracycline dose were detected. However, the same trend could not be confirmed when arterial and ventricular elastance, expression of CV reserve capacity (CVRC) [10], were evaluated. Therefore, 3D LVAC may represent a precocious marker of impaired cardiac performance, considering its significant correlation with endothelial function, cumulative anthracycline dose and time after chemotherapy exposure. Nevertheless, our data still underscore the necessity to find early modifications in CV physiology that can predict adverse long-term events.

Limitations should be acknowledged: the small sample-size, deriving from a previously published analysis, limits the generalization of our results. Hence, all conclusions should be considered hypothesis-generating. We also decided to exclude patients exposed to radiotherapy, that may have important effects on arterial and ventricular performance and may prevent a clear interpretation of direct anthracycline effects on the CV system. Second, in our cohort the lack of CV events or availability of cardiac biomarkers prevents attribution of a prognostic role to LVAC values. The follow-up of our patients was limited and therefore we cannot exclude that some events may be found at a later stage. Finally, we did not perform endothelial-independent estimation of brachial vasodilatation.

Limitatations should be weighted with our study’s strengths: this is the first report investigating LVAC, obtained by multiple echocardiographic methods, in CCSs, and correlating results with endothelial function. Our results pave the way to future directions: the study of ventricular-vascular coupling, both at rest and during exercise echocardiography may represent a new field of imaging in cardio-oncology, not only in CCSs but also in adults undergoing recently-introduced therapies. Nevertheless, considering the acknowledged limitations, verification of our findings in larger populations, in particular with high comorbidity burden, is hence warranted. Overall, our study highlights the critical need for more detailed characterisation of arterial properties and their impact on outcomes in cardio-oncology.

## 5. Conclusions

Echocardiographic LVAC and its correlation with endothelial function have never been investigated in survivors of childhood malignancies [36]. In our population, previously exposed to anthracycline therapy, we could not detect significant alterations in terms of arterial and ventricular elastance estimated by echocardiography, as well as of endothelial function. However, 3D SV and consequently 3D ESV/SV were significantly impaired in CCSs when compared to controls, this last one with a positive correlation with chemotherapy cumulative doses and years after exposure. Whether these parameters may portend for clinical implications over longer follow-up deserve careful assessment in large prospective studies.

## Data Availability

All data used or analysed during the current study are available from the corresponding author on reasonable request.
